# An introduction to overviews of reviews: planning a relevant research question and objective for an overview

**DOI:** 10.1186/s13643-018-0695-8

**Published:** 2018-03-01

**Authors:** Harriet Hunt, Alex Pollock, Pauline Campbell, Lise Estcourt, Ginny Brunton

**Affiliations:** 1 Exeter Test Group and PenCLAHRC, University of Exeter Medical School, St Luke’s Campus, Exeter, Devon EX1 1TE UK; 2 Nursing Midwifery and Allied Health Professions (NMAHP) Research Unit, Glasgow Caledonian University, Cowcaddens Road, Glasgow, G4 0BA UK; 3 Nursing Midwifery and Allied Health Professions (NMAHP) Research Unit, Glasgow Caledonian University, Cowcaddens Road, Glasgow, G4 0BA UK; 4 NHS Blood and Transplant, Oxford and Radcliffe Department of Medicine, University of Oxford, Level 2, John Radcliffe Hospital, Oxford, OX3 9BQ UK; 5 UCL Institute of Education, University College London, 20 Bedford Way, London, WC1H 0AL UK

**Keywords:** Overview, Systematic review, Evidence synthesis

## Abstract

**Background:**

Overviews of systematic reviews are a relatively new approach to synthesising evidence, and research methods and associated guidance are developing. Within this paper we aim to help readers understand key issues which are essential to consider when taking the first steps in *planning* an overview. These issues relate to the development of clear, relevant research questions and objectives prior to the development of an overview protocol.

**Methods:**

Initial discussions and key concepts for this paper were formed during a workshop on overview methods at the 2016 UK Cochrane Symposium, at which all members of this author group presented work and contributed to wider discussions. Detailed descriptions of the various key features of overviews and their different objectives were created by the author group based upon current evidence (Higgins J, Green S. Cochrane Handbook Syst Rev Interv. 2011;4:5, Pollock M, et al. Sys Rev. 2016;5:190-205, Pollock A, et al. Cochrane overviews of reviews: exploring the methods and challenges. UK and Ireland: Cochrane Symposium; 2016, Pieper D, et al. Res Syn Meth. 2014;5:187–99, Lunny C, et al. Sys Rev. 2016;5:4-12, Hartling L, et al. Comparing multiple treatments: an introduction to overviews of reviews. In 23rd Cochrane Colloquium; 2015, Hartling L, et al. Plos One. 2012;7:1-8, Ballard M, Montgomery P. Res Syn Meth. 2017;8:92-108) and author experiences conducting overviews.

**Results:**

Within this paper we introduce different types of overviews and suggest common research questions addressed by these overviews. We briefly reflect on the key features and objectives of the example overviews discussed.

**Conclusions:**

Clear decisions relating to the research questions and objectives are a fundamental first step during the initial planning stages for an overview. Key stakeholders should be involved at the earliest opportunity to ensure that the planned overview is relevant and meaningful to the potential end users of the overview. Following best practice in common with other forms of systematic evidence synthesis, an overview protocol should be published, ensuring transparency and reducing opportunities for introduction of bias in the conduct of the overview.

## Background

It is estimated that around 22 new systematic reviews are published every day [1]. In order to keep pace with the increasing volume of reviews, new methodological approaches have been developed for synthesising this evidence including overviews (systematic reviews of systematic reviews). Overviews are most frequently employed where multiple systematic reviews already exist on similar or related topics, and aim to systematically bring together, appraise and synthesise the results of related systematic reviews. Overviews have evolved to address a growing need to filter the information overload, improve access to targeted information and inform healthcare decision-making [2, 3]. Overviews can be useful tools to support decision-making by clinicians, policy makers and developers of clinical guidelines [2, 4]. There are a range of factors to reflect upon prior to deciding whether to conduct an overview, including consideration of the methodological challenges and uncertainties. These challenges are discussed in depth in our accompanying paper on this topic [5].

Overviews are known by a variety of different names, all potentially reflecting different aspects and aims of the syntheses. Terms used include: overview; umbrella review; meta-review; (systematic) review of (systematic) reviews; synthesis of systematic reviews; and summary of systematic reviews. The common feature of the methods associated with all of these terms is the fundamental process of synthesising evidence which is derived, often exclusively, from systematic reviews. The systematic review forms the primary ‘unit of analysis’ and is the basis upon which an overview is built [6].

The term ‘overview of systematic reviews’ (often shortened to ‘overview’) has gained widespread acceptance, and is the term used by Cochrane to describe a review of systematic reviews published in the Cochrane Library [7]. We use the term ‘overview’ within this paper to describe systematic summaries of systematic review evidence, in line with the most commonly used terminology.

Overviews can play a role in signposting the reader to evidence, summarising existing research or highlighting the absence of evidence [7]. For this reason, overviews can provide an ‘entry point’ for policy makers and other consumers by summarising broad issues and current knowledge around a topic, and directing the reader to more detailed, fine-grained material contained in component systematic reviews and primary research [8–10].

Likewise, the involvement of stakeholders at an early point in planning and conducting an overview may help in shaping these aims for maximum overview impact [2, 11, 12].

Overviews arguably have a valuable role where evidence relating to a specific topic exists but is conflicting, bringing together reviews in a transparent and systematic way and aiding informed decision making by gathering, appraising and systematically analysing this evidence. While the evidence synthesised within an overview may be used to generate new insights and understanding, it is important to note that overviews are fundamentally a method of bringing together, summarising and enhancing accessibility of existing evidence. 

## Methods

Overviews are a relatively new and emerging method of summarising evidence, and consequently universally-accepted guidance for good practice relating to the conduct of overviews is currently lacking [5, 13–17]. During a 2016 UK Cochrane Symposium workshop [18] focused on the methods and challenges associated with overviews, it became apparent that there was a need to clarify, and distinguish between, different types of overviews and the objectives which these overviews addressed. Within this paper, we therefore describe types of overview and the common research questions and objectives they address. Within a second, linked paper [5], we build on this description of overview types, objectives and research questions, illustrating this through the use of five exemplar overviews, and exploring the impact and implications of different methodological approaches.

In presenting and discussing common research questions addressed by overviews with different objectives, and relating this to real examples in the second paper [5], we aim to help readers understand important issues to consider during the first steps to planning an overview.

### Research questions and objectives addressed by overviews

In common with all research, overviews are carried out to address a clearly-stated research question. When planning an overview, determining the nature of the initial research question, and identifying who is asking the question, will dictate the scope of the overview objective(s). The objectives of an overview may include summarising existing evidence on a range of different topics, including: interventions; diagnostic accuracy of medical tests or procedures; prognosis or risk prediction; health equity [19]; or more qualitative aspects associated with any of the above, such as patient preference or device acceptability. In addition to summarising the results of multiple systematic reviews on related topics, overviews may also be used to investigate different aspects of questions already tackled by existing systematic reviews, such as variations in population, condition or intervention [10–12]. One example of this latter approach is provided in an overview which aimed to synthesise current evidence of the relationship between sedentary behaviour and health outcomes [20], reporting variation in results across populations and condition studied.

The principles which guide development of focussed clinical questions for systematic reviews remain valid for the development of research questions for overviews. Clearly defining the target population and setting, context, intervention, index test or phenomenon of interest, comparator or reference standard and outcome or treatment decisions are all essential parts of any overview protocol. The research question and overall overview objective will dictate the ‘type’ of overview that is required. This may be an overview of specific types of systematic review, or of systematic reviews which contain specific types of primary research study.

These defining elements of research questions and objectives are illustrated in Table 1, and we consider the objectives of each overview type in more detail below.

**Table 1:** Forming an overview question and defining the type and objective of an overview

Focus of question	Question about the effect of intervention(s)		Question about diagnostic test accuracy	Question about prognosis/prevelence	Questions about disease aetiology or risk factors	Question about qualitative views or experiences
Population	Is the focus on one discrete population, or bringing together evedence from different populations?					
Intervention/phenomenon of interest	Is the focus on one specific intervention or a range of different possible interventions?		Is the focus on one single medical test or a range of different medical tests?	Is the focus on overall prognosis, prognostic factors or risk prediction (prognostic models)?	Is the focus on associations between disease indices and risk factors, or the impact of risk factors on single/multiple outcomes?	Is the focus on qualitative aspects of an intervention, such as views or experiences of a particular group of people?
Outcome	Is the focus on one specific outcome (including adverse events) or a range of different outcomes?					
Context/setting	Describe the context of setting of interest					
Type of review	Review of RCTs	Review of intervention studies	Reviews of diagnostic test accuracy studies	Review of prognosis/prevalence	Reviews of associated risk factors	Review of qualitative studies
Type of overview	Overview of intervention reviews		Overview of diagnostic test accuracy reviews	Overview of reviews of prognostic studies	Overview of reviews of risk factors	Overview of qualitative reviews
Overview objective	To summarise evidence from more than one systematic review of different interventions for the same condition or problem; OR		To summarise evidence from more than one systematic review of diagnostic test accuracy assessing the same medical test to address the same condition of problem; OR	To summarise evidence about prognosis/prevalence from more than one systematic reviews.	To summarise systematic review evidence about risk factors not directly aligned to predictive values.	To summarise systematic review evidence relating to qualitative views or experiences.
	To summarise evidence from more than one systematic review of the same intervention for the same condition of problem where different outcomes are addressed in different systematic reviews; OR		To summarise evidence from more than one systematic review of diagnostic test accuracy assessing different medical tests to address the same condition or problem.			
	To summarise evidence from more than one systematic review of the same intervention for different conditions, problems or populations; OR					
	To summarise evidence about adverse effects of an intervention from more than one systematic review of use of the intervention for one or more conditions.					

Adapted from Becker LA, Oxman AD. Chapter 22: Overviews of reviews. In: Higgins JPT, Green S (editors). Cochrane Handbook for Systematic Reviews of Interventions. Version 5.1.0 [updated March 2011]. The Cochrane Collaboration, 2011. Available from http://training.cochrane.org/handbook and Deeks JJ, Bossuyt PM, Gatsonis C (editors), Cochrane Handbook for Systematic Reviews of Diagnostic Test Accuracy Version 0.9. The Cochrane Collaboration, 2013. Available from: http://methods.cochrane.org/sdt/handbook-dta-reviews

## Overviews of intervention reviews

Overviews of intervention reviews should be considered when the research question relates to the effectiveness of one or more intervention. Common objectives for overviews of intervention reviews are detailed below.

### To summarise evidence from more than one systematic review of different interventions for the same condition or problem

This is the primary purpose of Cochrane Overviews of Interventions, and a number of overviews of interventions have been employed to tackle this objective [21–23].

For example, one overview has brought together all systematic reviews of interventions to improve arm function in people with stroke [22], whilst another overview has summarised systematic reviews of conservative interventions for the treatment of incontinence in women [21]. One example of a mixed methods overview assessed all workplace health promotion interventions using healthcare or wellbeing outcomes from systematic reviews of effectiveness, combining these with syntheses of identified policy documents and research on stakeholders’ perspectives of workplace intervention programmes [23].

### To summarise evidence from more than one systematic review of the same intervention for the same condition or problem where different outcomes are addressed in different systematic reviews

Overviews of interventions can be used to summarise evidence assessing different outcomes for the same condition. Generally, systematic reviews should include all outcomes that are important to people making decisions about and influenced by an intervention. This includes the involvement of stakeholders in order to reflect aspects important to people receiving an intervention [24], and should be incorporated at the study, review and overview levels. Not all systematic reviews, however, focus on a single condition or outcome. For example, one overview has brought together all systematic reviews of the use of red cell transfusion to prevent or treat common complications in people with sickle cell disease, such as painful crises, stroke and acute chest syndrome [25]. This overview assessed the prevention of these complications during high-risk situations such as surgery, pregnancy or a sub-population identified as at high risk of a particular complication, such as abnormal transcranial Doppler and risk of stroke in children. Another overview summarised the safety of long-acting beta agonists (regular formoterol or salmeterol) in children with asthma with outcomes including all-cause mortality, non-fatal serious adverse events, asthma-related deaths and asthma-related non-fatal serious events [26]. This overview was prompted by concerns raised by two large surveillance studies in adults with asthma [27, 28] that found an increased risk of asthma-related mortality in those who took regular salmeterol and the weaker evidence base for the effectiveness of long-acting beta agonists in children.

### To summarise evidence from more than one systematic review of the same intervention for different conditions, problems or populations

The same or similar interventions are often used for different conditions or different studies and reviews may focus on different populations. This type of evidence may be of interest where more than one patient population is being addressed, as generalisability of the effect may be more extensive. One recent example is of an overview of reviews of cognitive rehabilitation for different cognitive problems in people with stroke [29].

### To summarise evidence about adverse effects of an intervention from more than one systematic review of use of the intervention for one or more conditions

Systematic reviews often report information on adverse effects, but few reviews are conducted with the main aim to report rates of these events. This may well change following the recent publication of the PRISMA harms checklist [30]. Due to the rarity of many adverse events, randomised controlled trials rarely contain sufficient data to give an accurate indication of prevalence [31, 32]. It would therefore be inappropriate to rely on systematic reviews based solely on trial data to profile adverse events of a specific intervention, except in the rare situations where the recording of adverse effects data is the primary aim of the trial [33]. It may be appropriate to include data not previously included in a systematic review such as when conducting a Health Technology Assessment (HTA) report, developing a clinical practice guideline or developing resources such as *BMJ Clinical Evidence*. One overview summarising evidence on adverse effects of herbal medicines across all conditions takes this broader approach to systematic review evidence and provides an example of methodological challenges encountered [34].

## Overviews of diagnostic test accuracy reviews

Overviews of diagnostic test accuracy reviews should be considered when the research question relates to the accuracy of one or more diagnostic test. Common objectives for overviews of diagnostic test accuracy reviews are addressed below.

### To summarise evidence from more than one systematic review of diagnostic test accuracy assessing the same medical test to address the same condition or problem

The purpose of diagnostic test accuracy overviews is to form a summary of systematic review evidence in order to address a specific research question, where the unit of interest is systematic reviews of diagnostic test accuracy. These systematic reviews are designed to assess existing evidence of the diagnostic accuracy of a test or device using standard measures of accuracy (sensitivity and specificity) rather than measures of effectiveness as with reviews of interventions. Systematic reviews of diagnostic test accuracy commonly encounter greater heterogeneity than intervention reviews, due to variation in study populations, in the testing environment and context, or in procedures used to conduct the tests involved [35]. Overviews of diagnostic test accuracy which aim to assess the accuracy of a single medical test generally have more potential for identifying sources of heterogeneity than overviews which address a number of additional variables such as multiple tests or devices [35]. An example in which this approach has been taken is the overview of systematic review evidence of the diagnostic accuracy of endoscopic ultrasonography (EUS) for the preoperative loco-regional staging of primary gastric cancer [36]. The authors reported that substantial heterogeneity may have influenced the applicability of clinical usefulness for endoscopic ultrasonography for pre-operative loco-staging of primary gastric cancer [36]. By undertaking an overview, the authors were able to identify the need for greater understanding of the sources of heterogeneity before recommendations could be made about the clinical usefulness of EUS. Overview authors were also able to make more nuanced practice recommendations on test performance, and this ability to pinpoint specific areas for further research as well as issue practice guidance demonstrates a potential benefit of overviews.

### To summarise evidence from more than one systematic review of diagnostic test accuracy assessing different medical tests to address the same condition or problem

Overviews assessing the diagnostic test accuracy of different medical tests addressing the same condition are similar in terms of scope and objectives to the overviews described in the previous section, with the key difference that a number of different medical tests are being assessed within included systematic reviews. For example, a recently-conducted overview summarising the diagnostic test accuracy of brief cognitive assessments for identifying dementia in a primary care population [37] included evidence from a range of systematic reviews of diagnostic test accuracy of a number of different brief cognitive assessments. This enabled conclusions to be drawn about the accuracy of specific tests within the primary care population, and signposted a gap in current evidence for direct comparisons of the diagnostic accuracy of individual tests for identifying dementia in primary care. Again, these broader recommendations were made possible through the wider synthesis of existing evidence than had previously been conducted in this specific setting.

## Overviews of reviews of prognosis/prevalence

Overviews of reviews of prognosis/prevalence should be considered when the objective is to summarise evidence about prognosis/prevalence from more than one systematic review. The implementation of overview methodology in this field is relatively recent, but there are a growing number of systematic reviews specifically investigating the predictive value of tests and devices, prognostic information and/or prognostic models. These address questions such as ‘what is the most likely course of this health condition?’ ‘What factors are associated with outcome?’ and ‘are there risk groups likely to have different outcomes?’ [38]. One example of such an overview evaluated the prognostic evidence alongside evidence on treatment, harms, diagnosis, classification and outcomes used for managing neck pain [39].

## Overviews of reviews of risk factors

These overviews incorporate disease aetiology or risk factors when the risks of interest may not directly relate to prognostic variables or risk prediction models. When planning to conduct an overview of systematic review evidence in order to explore the effect of putative risk factors on a range of variables, factors to consider include whether the primary interest in conducting the overview is to explore associations between markers of a disease and known risk factors, or whether the main focus is the impact of those risk factors on single or multiple outcomes. An example overview addressing the latter purpose is an overview which aimed to evaluate the strength and validity of the evidence for the association between adiposity and risk of developing or dying from cancer [40]. The authors of this work found strong evidence of an association between obesity and 11 of the 36 studied cancer sites and subtypes. The cancers for which there was strong evidence of an association with obesity were mainly cancers of digestive organs and female hormone-related malignancies. The authors of the overview concluded that whilst other associations could be genuine, substantial uncertainty remains for the other cancers studied.

## Overviews of qualitative reviews

Overviews of reviews of qualitative reviews should be considered when the objective is to summarise systematic review evidence relating to qualitative views or experiences. There is clear guidance available on the good conduct of an overview of qualitative syntheses [6], with commonalities across all types of overviews. Common features include employing an a priori peer-reviewed protocol formed around a clearly pre-specified research question with detailed inclusion and exclusion criteria, search strategies and methods for data extraction and appraisal, followed by clear and replicable methods for synthesis and summary of included data [6]. An example overview using qualitative data as well as quantitative information is provided by an overview exploring improving quality of care for persons with diabetes looking at a broad range of interventions, including patient education and support, telemedicine, organisational changes and outcomes relating to the process of care [10]. In combining these approaches, overview authors had potential to synthesise data on patient experiences of quality of care alongside quantitative evaluation of effectiveness, which could result in a richer set of evidence for informing practice and policy.

Whilst many overviews tacitly assess quantitative outcomes reported in systematic reviews [6], often the nature of overviews results in narrative synthesis which can draw on either quantitative or qualitative data within included systematic reviews. In this sense, many overviews include elements of qualitative data identified within the source systematic reviews.

## Results and Discussion

There are many similarities between overviews and systematic reviews, and the principles which guide the planning of a systematic review (including production of a clinically-relevant research question and a pre-specified peer reviewed protocol) are relevant in conducting an overview [2]. Within this paper, we have described a brief classification to organise common research questions and objectives, using overviews based on frameworks developed within the Cochrane Handbooks for Systematic Reviews of Interventions [41] and Diagnostic Test Accuracy [42]. These descriptions cover overviews of intervention reviews, overviews of diagnostic test accuracy reviews, overviews of reviews of prognosis/prevalence, overviews of reviews of risk factors and overviews of reviews of qualitative studies.

Overviews aim to summarise evidence and to signpost readers to relevant sources to support decision making; this paper has highlighted that there are a wide range of potential reasons for selecting to do an overview, and that these varied reasons lead to overviews which may have a number of different methodological features.

Overviews of reviews of different interventions for the same condition, or of the same intervention but looking at different outcomes, will have high clinical relevance where clinical decisions are made between different treatments. Overviews of intervention reviews, bringing together evidence relating to the effectiveness of a specific treatment applied in alternative populations or settings will be of interest to healthcare providers delivering that treatment, or to consumers seeking information about the effective interventions. Overviews of risk factors will have similar clinical interest and potential relevance for policymakers and regulators. Overviews relating to the adverse effects of an intervention in the same or different conditions may allow commonalities to be drawn across a broader range of evidence than in a more focussed systematic review, with the potential to highlight equivalence or patterns not previously identified. Similarly, overviews of systematic reviews of diagnostic test accuracy provide an opportunity to gain greater insights into test accuracy data summarised across different populations, settings or other variables, with potential to reduce the impact of data heterogeneity by drawing on a broader evidence base. Overviews of prognosis are also increasing in number and scope, offering potential to provide useful insights by summarising evidence of the likely course of a condition, factors associated with health outcomes or identifying risk groups associated with different health outcomes [38] . When applied within systematic frameworks, overviews of qualitative evidence provide scope for creating theoretically-defined conceptions of complex topics [43].

Often, the scope of systematic reviews can be described as either ‘lumping’ or ‘splitting’ information [44, 45]. Lumping refers to finding commonalities across different approaches, whereas splitting creates a more narrowly-refined focus within a broader research field. Systematic reviews of primary research often split data by addressing a focussed and specific research question which may not be very useful for informing broader clinical and policy decision making. Conversely, overviews commonly adopt a ‘lumping’ approach, allowing greater leeway for generality in research findings [45], and arguably having greater applicability for policy makers. There are clearly challenges in ‘lumping’ large volumes of information, and presenting this in an accessible format, which is relevant and useful to the end user. Another significant challenge in lumping information is how to consistently synthesise such information in the face of inevitable heterogeneity.

The classification we have employed here suggests a range of common objectives and research questions which may be addressed by an overview, where the primary objective is to summarise the existing body of systematic review evidence on a topic. The scope of this summary of evidence is defined by previously stated inclusion and exclusion criteria [6, 13]. This summary of evidence should not simply duplicate the reporting of individual systematic review summaries, but instead should aim to synthesise across included systematic review evidence in order to bring new insights to existing evidence. The suitability of reanalysis of existing data within an overview is debated, and it has been argued that, where novel analyses are the aim, conducting a review of trials may be more appropriate than an overview of reviews [14]. Methodological guidance on the reporting of systematic reviews using individual participant data has been published by the PRISMA-IPD Group [46] and may prove relevant to reporting within overviews which aim to incorporate novel analyses. It is clearly important for the stated overview research questions and objectives to specify any plans for data analysis, and for this to be planned with reference to the available methodological guidance, and with appropriate justification of the use of any overview of reviews, rather than a review of trials.

At its broadest sense, the common purpose of an overview is to provide an accessible summary of evidence, in order to support decision making by clinicians, policy makers and developers of clinical guidelines [2]. It is now widely accepted that in order to ensure relevance and impact of health research, key stakeholders (including but not restricted to people with a healthcare condition, their families, friends and caregivers, health professionals and decision makers) should be involved in the process [47, 48]. Central to the conduct of an overview are the people involved in its production. From formulating the question to conducting the overview and disseminating findings, the specific purpose of an overview may change depending on who is asking the research question and clearly stakeholders should be actively involved throughout the process. The involvement of key stakeholders, including patients and their families or carers, should occur at the earliest opportunity in order to ensure that the planned overview is relevant and meaningful to the potential end users of the overview.

## Conclusions

Overviews are a relatively new methodological approach and consequently a number of aspects of overview methodology remain uncertain. It is the responsibility of a research team to decide on their approach before conducting an overview; central to this is determining what type of overview is to be conducted. Clear decisions relating to the research questions and objectives to be addressed by the overview are a fundamental first step during the initial planning stages for an overview, and should be developed with the involvement of key stakeholders. Following best practice, these aspects should be covered within a published overview protocol as a mechanism for ensuring transparency and reducing opportunities for introduction of bias in the conduct of the overview. Our second paper [5] outlines a number of key methodological decisions which we consider important to address when planning an overview, and which will be important to incorporate within an overview protocol.

Despite a need for improved guidance for the conduct of overviews [2], there are a number of resources available which support the conduct of overviews [2, 6, 7, 13], and updates to the relevant chapter of the Cochrane Handbook are currently in production [7]. Further guidance on the less common types of overview (such as those addressing reviews of diagnostic tests accuracy and prognosis) and more challenging aspects of overview production, such as methods for narratively synthesising findings, dealing with missing data, poor reporting and dealing with complexity versus granularity [10], would be a great benefit to those tackling overviews. In the absence of empirical evidence to support the selection and implementation of overview methods, we believe that the use of illustrated examples of real-life overviews will be helpful to authors planning new overviews, and to those seeking to establish evidence relating to optimal overview methods. This is therefore the focus of our second paper on this topic [5].

## Abbreviations

BMJ: British Medical Journal
HTA: Health Technology Assessment
PRISMA: Preferred Reporting Items for Systematic Reviews and Meta-Analyses [systematic review reporting guidelines]
PRISMA-IPD: Preferred Reporting Items for Systematic Reviews and Meta-Analyses – Individual Patient Data
PROSPERO: International Prospective Register of Systematic Reviews
RCTs: Randomised Controlled Trials
